# Amelioration of IFN-γ and TNF-α-Induced Intestinal Epithelial Barrier Dysfunction by Berberine via Suppression of MLCK-MLC Phosphorylation Signaling Pathway

**DOI:** 10.1371/journal.pone.0061944

**Published:** 2013-05-03

**Authors:** Min Cao, Pei Wang, Chunhong Sun, Wen He, Fengjun Wang

**Affiliations:** State Key Laboratory of Trauma, Burns and Combined Injury, Institute of Burn Research, Southwest Hospital, Third Military Medical University, Chongqing, China; University of Chicago, United States of America

## Abstract

Intestinal barrier dysfunction occurs in many intestinal diseases, in which proinflammatory cytokines play critical roles. However, researchers are still on the way to defining the underlying mechanisms and to evaluate therapeutic strategies for restoring intestinal barrier function. Berberine, a drug that has clinically been used to treat gastroenteritis and diarrhea for thousands of years, has been shown to protect barrier function in both endothelial and epithelial cells, but the mechanisms are completely unknown. In this study, we investigate the protective actions of berberine on barrier function and the underlying mechanisms in Caco-2 monolayers challenged with IFN-γ and TNF-α. Caco-2 monolayers were treated without or with simultaneous IFN-γ and TNF-α in the absence or presence of berberine. Both transepithelial electrical resistance (TER) and paracellular permeability were measured to evaluate barrier function. The expression and distribution of tight junction proteins ZO-1, occluding, and claudin-1 were respectively analyzed by immunoblot or immunofluorescence. The expressions of phosphorylated myosin light chain (pMLC), MLC kinase (MLCK) and hypoxia-inducible factor-1α (HIF-1α) were determined by immunoblot. The translocation of NF-κB p65 to nuclei was analyzed by immunofluorescence and immunoblot, respectively. The results showed that berberine significantly attenuated TER decrease and paracellular permeability increase in Caco-2 monolayers treated with IFN-γ and TNF-α. Berberine also dramatically alleviated IFN-γ and TNF-α-induced morphological alteration of tight junction proteins ZO-1, occluding, and claudin-1. The increase of both MLC phosphorylation and MLCK protein expression induced by IFN-γ and TNF-α was significantly inhibited by berberine treatment. Additionally, berberine suppressed the activation of HIF-1α, but not NF-κB. Taken together, it is suggested that berberine attenuates IFN-γ and TNF-α-induced intestinal epithelial barrier dysfunction by inhibiting the signaling pathway of MLCK-dependent MLC phosphorylation mediated by HIF-1α.

## Introduction

It is well known that an intact intestinal epithelial barrier plays an important role in preventing luminal pathogens and antigenic molecules from coming into the intestinal mucosa and contacting with the immune system, and that tight junction and its associated proteins, such as zonula occludens (ZO), occludin and claudins, are critical to the maintenance of the intact intestinal epithelial barrier [1]–[4]. However, the intestinal epithelial barrier function is frequently disrupted in a variety of acute or chronic enteropathies, such as inflammatory bowel disease, irritable bowel syndrome, and infectious diarrhea [4]–[7]. During the process of these enteropathies, many proinflammatory cytokines are released within the intestinal mucosa. These proinflammatory cytokines including interferon (IFN)-γ, tumor necrosis factor (TNF)-α, interleukin (IL)-1β, IL-6, IL-13 and TNF superfamily member LIGHT have been documented to contribute to the disruption of intestinal epithelial barrier function [4], [8]–[15]. Although the underlying mechanisms are incompletely understood, it has been believed that myosin light chain (MLC) phosphorylation mediated by MLC kinase (MLCK) plays a very important role in the proinflammatory cytokines-induced intestinal barrier disruption [9]–[11], [13], [15].

Although the compromised disruption of intestinal barrier function may be either causative or consequential, it has been proposed to play a very important role in the pathogenesis and relapse of inflammatory bowel disease including Crohn’s disease and ulcerative colitis [16]–[18]. In addition, it has been documented that primary pathophysiologically relevant intestinal epithelial barrier dysfunction can broadly activate mucosal immune responses and accelerate the onset and severity of immune-mediated colitis [19]. Thus, restoring the disrupted intestinal barrier function is beneficial for eliminating or alleviating the mucosal inflammation and immune responses.

Berberine is one of the main constituents of *Coptidis rhizome* that has widely been used as a traditional drug to treat gastrointestinal disorders such as gastroenteritis and diarrhea for thousands of years in China. Berberine has so far been viewed as a drug with pleiotropic biochemical and pharmacological effects, including anti-inflammatory, anti-bacterial, anti-parasitic, anti-oxidatic, anti-apoptotic, and anti-tumor actions [20]–[25]. In addition, some previously published *in vivo* studies have demonstrated that berberine ameliorates experimental colitis induced by either trinitrobenzene sulfonic acid or dextran sulfate sodium in mice or rats [23], [26]–[28], which is largely attributed to the anti-inflammatory properties of berberine. It has also been reported that berberine protects barrier function in both endothelial and epithelial cells [29]–[32]. However, the molecular mechanisms involved in the protective effects of berberine on barrier function are incompletely clear, and remain to be elucidated.

In this investigation, we examined the actions of berberine on barrier function and the underlying mechanisms in an *in vitro* model of human intestinal epithelia exposed to proinflammatory cytokines IFN-γ and TNF-α. Our data provided the direct evidence that berberine could attenuate intestinal epithelial barrier disruption induced by simultaneous IFN-γ and TNF-α. Additionally, our data revealed that berberine attenuated IFN-γ and TNF-α-induced intestinal epithelial barrier dysfunction via inhibition of MLCK-dependent MLC phosphorylation mediated by hypoxia inducible factor (HIF)-1α.

## Materials and Methods

### Cell Culture

Human colonic Caco-2 epithelial cell lines obtained from American Type Culture Collection (Manassas, VA) were grown in DMEM media (Invitrogen, Carlsbad, CA) supplemented with 10% fetal bovine serum, 4.0 mM L-glutamine, 1% non-essential amino acids, 100 U/ml penicillin, and 100 µg/ml streptomycin. Caco-2 cells were maintained in a humidified 37°C, 5% CO_2_ incubator, and passaged by partial digestion with 0.25% trypsin and 0.53 mM EDTA in Ca^2+^-free and Mg^2+^-free Hank’s balanced saline solution (HBSS).

### Monolayer Preparation and Treatment

To prepare Caco-2 monolayers, cells were plated at 5×10^4^/cm^2^ on collagen-coated permeable polycarbonate membrane Transwell supports with 0.4 µm pores (Corning, Corning, NY) and grown as monolayers for 14 days prior to experiments. For the experiments, monolayers were treated with recombinant human IFN-γ (10 ng/ml) and TNF-α (10 ng/ml) (R&D Systems, Minneapolis, MN) for 48 hours, without or with 100 µM berberine (Sigma, St. Louis, MO). Both cytokines and berberine were added to the basal chamber without manipulating the apical DMEM media. The monolayers incubated with DMEM media were used as control.

### Assessment of Transepithelial Electrical Resistance

Transepithelial electrical resistance (TER) as an indicator of tight junction permeability to ionic solutes were measured in all experimental Caco-2 cell monolayers using a Millicell-ERS voltohmmeter (Millipore, Bedford, MA) at each time point during the experiment. Each TER measurement was calculated by subtracting the resistance value of the filters and fluids. In order to facilitate comparisons between conditions, TER was normalized to the initial value.

### Paracellular Permeability Assay

Flux of fluorescein isothiocyanate (FITC)-conjugated dextran (FITC-dextran, 4 kDa, Sigma) across Caco-2 monolayers was used to stand for the paracellular permeability of intestinal epithelial barrier to uncharged macromolecules. FITC-dextran flux was determined as described previously [33], [34]. Briefly, monolayers were gently washed with HBSS and transferred to 500 µl HBSS. DMEM media in apical chamber were gently aspirated and replaced with 100 µl of 1 mg/ml FITC-dextran in HBSS. Then, monolayers were incubated at 37°C for 2 hours. 100 µl sample was taken from basal chamber and the fluorescence was determined using a fluorescent plate reader (Varioskan Flash, Thermo Electron Corporation, Vantaa, Finland) with an excitation wavelength of 480 nm and an emission wavelength of 520 nm. FITC-dextran flux was normalized to control.

### Immunoblot Analysis

Caco-2 cells grown on 5.0-cm^2^ Transwell supports were washed with ice-cold PBS, and lysed with Laemmli sample buffer (50 mM Tris-HCl, 2% SDS, 0.1% bromophenol blue, 5% β-mercaptoethanol, 10% glycerine, pH 6.80), followed by a brief sonication with a sonicator (Tomy Seiko, Tokyo, Japan). Cell lysates were centrifuged at 15,000 g for 10 min at 4°C and heated at 100°C for 5 min. Nuclear extracts were prepared with Nucbuster Protein Extraction kit (Novagen, WI) according to manufacturer protocol. Protein concentrations were determined by *RC DC* kit (Bio-Rad, Hercules, CA) in accordance with the manusfacture’s protocol. Proteins were separated on 10% SDS-PAGE gel and transferred to PVDF membrane (Millipore). After blocking with 5% non-fat milk for 1 hour at room temperature, membranes were incubated with primary antibodies specific for ZO-1(1∶1000, Invitrogen), occludin (1∶1000, Invitrogen), claudin-1 (1∶1000, Invitrogen), MLCK (1∶1000, Sigma), MLC (1∶1000, Sigma), phosphorylated MLC (pMLC, 1∶1000, Cell Signaling, MA), NF-κB p65 (1∶1000, Beyotime, Jiangsu, China), HIF-1α (1∶1000, Millipore), and β-actin (1∶5000, Sigma) overnight at 4°C. After washing, membranes were incubated with peroxidase-conjugated secondary antibodies (1∶5000, Southern Biotech, Birmingham, AL) for 1 hour at room temperature. The blots were developed with an enhanced chemiluminescence kit (GE Healthcare, Buckinghamshire, UK), and imaged using a ChemiDoc XRS system (Bio-Rad). Densitometric analysis was performed using Quantity One software (Bio-Rad).

### Immunofluorescence Microscopy

Caco-2 monolayers grown on 0.33-cm^2^ Transwell supports were washed with ice-cold PBS, fixed with 1% paraformaldehyde for 30 min, incubated with 50 mM NH_4_Cl for 15 min, and permeabilized with 0.1% Triton X-100 in PBS containing 1 mM CaCl_2_ at room temperature. Then, monolayers were blocked in 2.5% bovine serum albumin (BSA) and incubated with anti-ZO-1 (1∶200, Invitrogen), anti-occludin (1∶50, Invitrogen), anti-claudin-1 (1∶50, Invitrogen), or anti-NF-κB p65 (1∶300, Beyotime) antibodies diluted in PBS containing 5% BSA at 4°C overnight. Monolayers were washed with PBS followed by incubation with Alexa Fluor 488-conjugated anti-IgG (1∶50, Molecular Probes, Eugene, OR) or Texas red-conjugated anti-IgG secondary antibodies (1∶50, EMD Chemicals, Gibbstown, NJ) with DAPI (Biotium Inc, Hayward, CA) for 1 hour at room temperature. After washing with PBS, monolayers were mounted in Slowfade (Molecular Probes) and imaged using a laser scanning fluorescence microscopy (TCS SP5, Leica, Germany).

### Statistical Analysis

Differences among groups were performed using One-way ANOVA followed by Fisher’s Least Significant Difference test. Results are shown as the means ± SEM. A difference of *p*<0.05 was considered statistically significant. SPSS statistical software (version 13.0) was used for the statistical analysis.

## Results

### Berberine Attenuates Intestinal Epithelial Barrier Dysfunction Induced by IFN-γ and TNF-α

**Figure 1:**
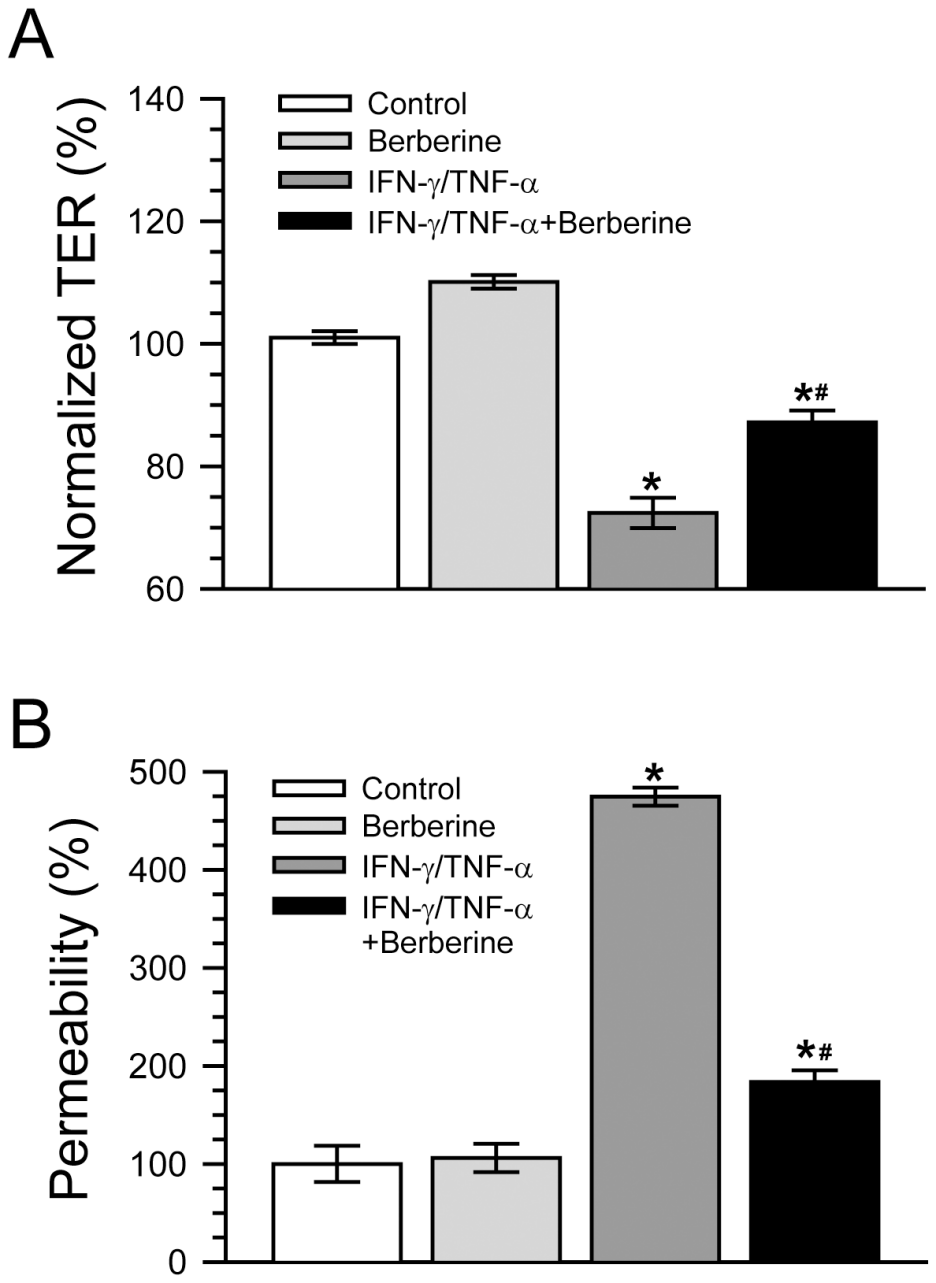
Berberine attenuates intestinal epithelial barrier dysfunction induced by IFN-γ and TNF-α. A. Caco-2 monolayers were incubated without or with 10 ng/ml IFN-γ and 10 ng/ml TNF-α in the absence or presence of 100 µM berberine for 48 h. Berberine significantly inhibited TER reduction induced by IFN-γ and TNF-α treatment. *p<0.05, compared with control, #*p*<0.05, compared with IFN-γ/TNF-α. n = 10. B. Caco-2 monolayers were treated as described in panel A. The IFN-γ and TNF-α-induced increase of paracellular permeability to 4 kDa FITC-dextran was significantly lowered by berberine treatment. *p<0.05, compared with control, #*p*<0.05, compared with IFN-γ/TNF-α. n = 7.

We, along with other investigators, have demonstrated that IFN-γ and TNF-α in combination disrupt intestinal barrier function *in vitro*, as evidenced by the decreased TER and the increased paracellular permeability [9], [33]–[35]. Thus, in order to investigate the effect of berberine on intestinal barrier function, we adopted an *in vitro* model in which human colonic Caco-2 epithelial cell monolayers were treated with simultaneous IFN-γ and TNF-α for 48 hours [33], [34]. Both TER, an indicator of epithelial paracellular permeability to ionic solutes, and dextran flux, an indicator of epithelial paracellular permeability to uncharged macromolecules, were employed to assess barrier function. As shown in Fig. 1A and B, berberine alone just caused a small increase in TER, but had no effect on paracellular permeability to 4 kDa FITC-dextran in control Caco-2 monolayers. The TER of Caco-2 monolayer treated with simultaneously IFN-γ and TNF-α for 48 hours was significantly lower than that of control monolayers (Fig. 1A), indicating that IFN-γ and TNF-α treatment increase the paracellular permeability to ionic solutes. However, berberine treatment significantly dampened the TER drop elicited by simultaneously IFN-γ and TNF-α (Fig. 1A).

Consistent with the above-mentioned changes of TER, the flux of 4 kDa FITC-dextran in Caco-2 monolayers treated with simultaneously IFN-γ and TNF-α for 48 hours was significantly higher than that of control monolayers (Fig. 1B), indicating that the paracellular permeability to nonionic macromolecules is increased by simultaneously IFN-γ and TNF-α treatment. Also as shown in Fig. 1B, berberine treatment significantly lowered the increase of paracellular dextran flux induced by simultaneously IFN-γ and TNF-α. These data suggest that berberine is capable of attenuating intestinal epithelial barrier dysfunction induced by IFN-γ and TNF-α *in vitro*.

### Berberine Prevents Morphological Disruption of Tight Junction Induced by IFN-γ and TNF-α

**Figure 2:**
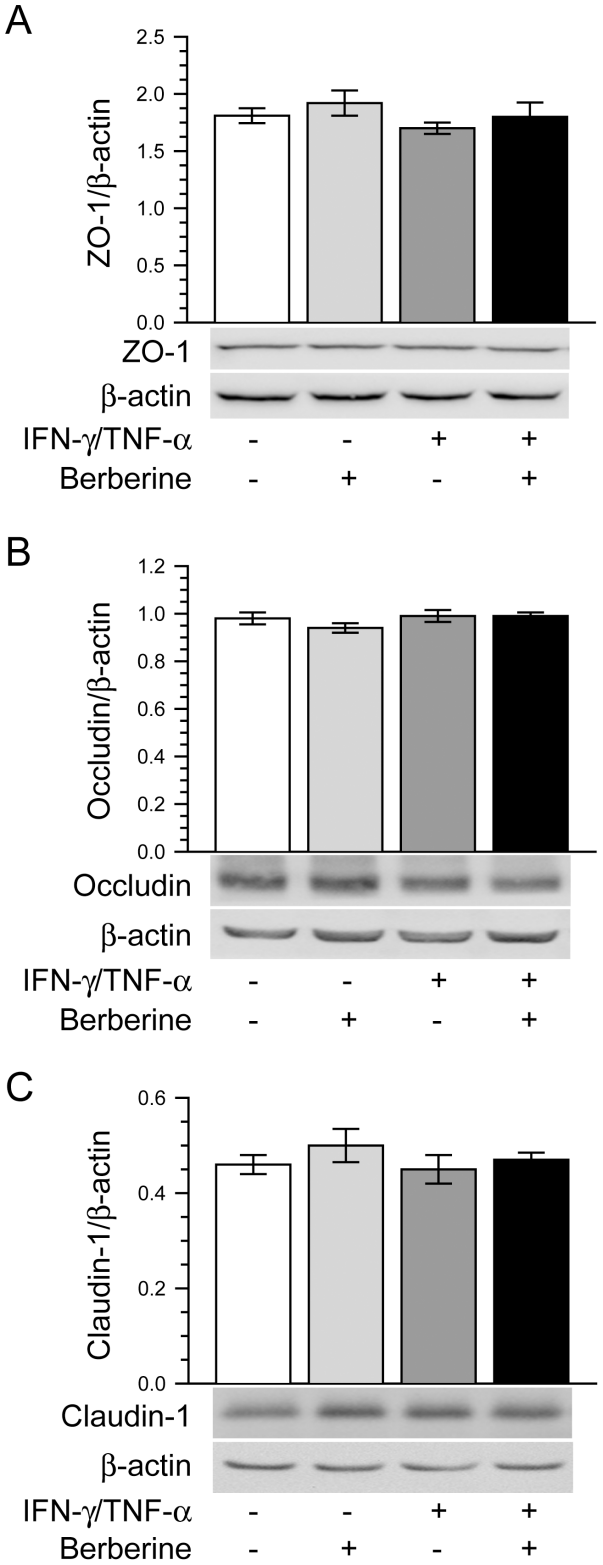
Berberine does not affect the expression of tight junction proteins. Caco-2 monolayers were treated as described in Fig. 1A. Cell lysates were analyzed to detect the expression of tight junction proteins ZO-1 (A), occludin (B) and claudin-1 (C) by immunoblot. The total protein expressions of cellular tight junction proteins ZO-1, occludin and claudin-1 were not significantly altered by the treatment of Caco-2 monolayers without or with IFN-γ and TNF-α in the absence or presence of berberine. Data are representative of five similar experiments.

It has been reported that the alteration of tight junction protein expression is involved in the intestinal barrier disruption induced by proinflammatory cytokines [12], [36]. Thus, we examined the effect of berberine on the total expression of tight junction proteins ZO-1, occludin and claudin-1 in Caco-2 monolayers treated with or without IFN-γ and TNF-α. As shown in Fig. 2A, B, and C, the total protein expressions of cellular tight junction proteins ZO-1, occludin and claudin-1 were not significantly altered by the treatment of Caco-2 monolayers with or without IFN-γ and TNF-α in the absence or presence of berberine. This is similar to previous studies revealing that the protein expressions of tight junction proteins are unchanged in Caco-2 monolayers after IFN-γ and TNF-α challenge [9], [33].

**Figure 3:**
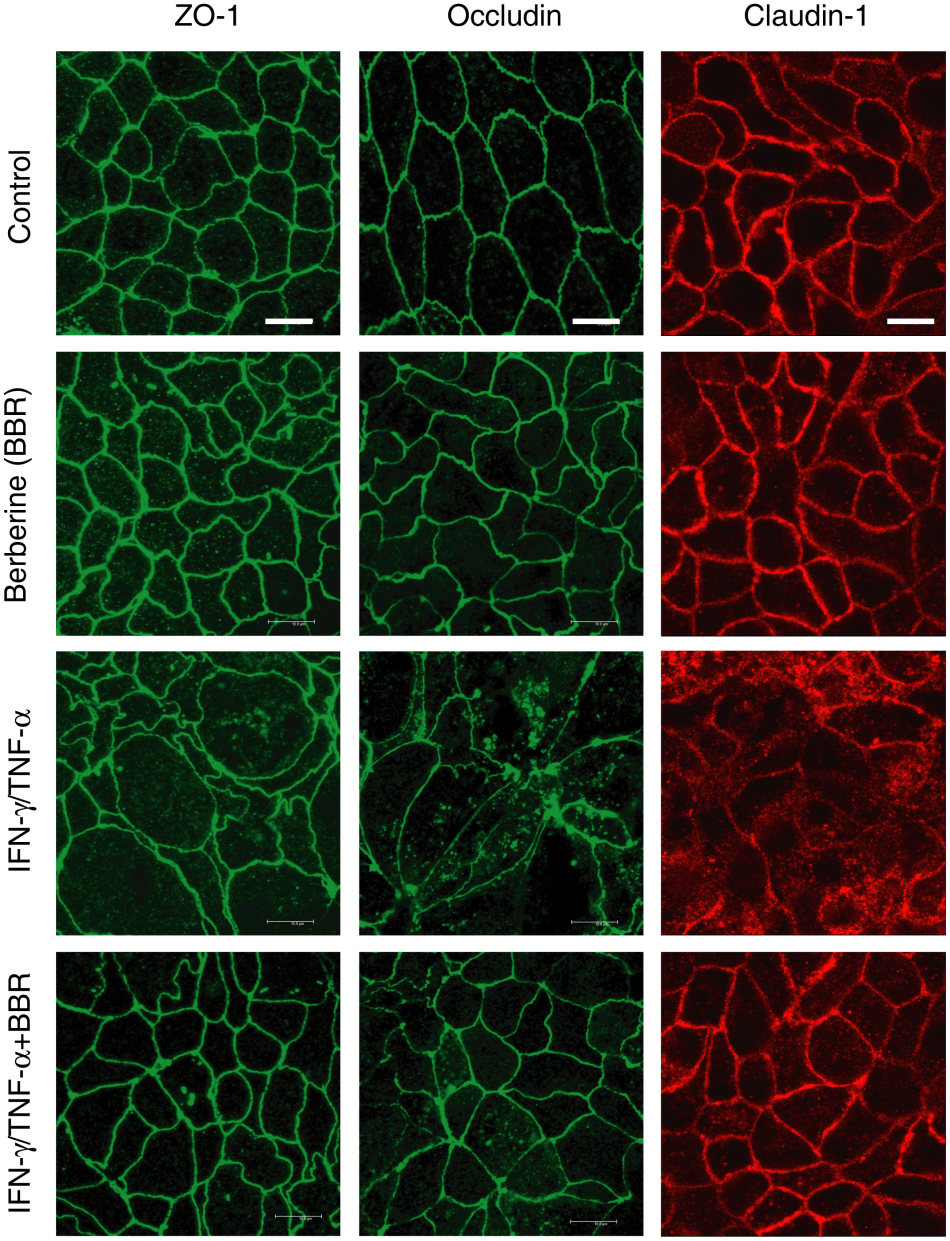
Berberine prevents morphological disruption of tight junction induced by IFN-γ and TNF-α. Caco-2 monolayers were treated as described in Fig. 1A. Tight junction proteins ZO-1, occludin and claudin-1 were stained by immunofluorescence. Berberine dramatically prevented the IFN-γ and TNF-α-induced morphological disruption of tight junction proteins ZO-1, occludin and claudin-1 in Caco-2 monolayers. Data are representative of four independent experiments. Scale bar = 10 µm.

The intestinal epithelial barrier function is regulated by tight junction structure. Previous studies have shown that intestinal barrier dysfunction induced by proinflammatory cytokines is associated with the morphological tight junction disruption and the relocalization of tight junction proteins [9], [10], [15], [33]–[35]. Thus, we next determined whether berberine affected the morphological localization of tight junction proteins in Caco-2 monolayers treated with or without IFN-γ and TNF-α. As illustrated in Fig. 3, in control Caco-2 monolayers, the tight junction proteins ZO-1, occludin and claudin-1 were respectively localized to the intercellular tight junctions, along the edge of the cells. These regular distributions were not obviously changed in Caco-2 monolayers treated with berberine alone for 48 hours. Treatment of Caco-2 monolayers with IFN-γ and TNF-α for 48 hours induced pronounced reorganization of tight junction proteins ZO-1, occludin and claudin-1 such that the distribution profiles became irregular and discontinuous. In addition, both occludin and claudin-1 were partially internalized into cytoplasmic vesicles, but ZO-1 internalization was not obviously seen. In contrast, berberine treatment largely attenuated the IFN-γ and TNF-α-caused reorganization of ZO-1, occludin and claudin-1 in Caco-2 monolayers. These indicate that berberine could prevent the reorganization of tight junction proteins induced by proinflammatory cytokines in intestinal epithelia.

### Berberine Inhibits IFN-γ and TNF-α-induced Increases of MLC Phosphorylation and MLCK Protein Expression

**Figure 4:**
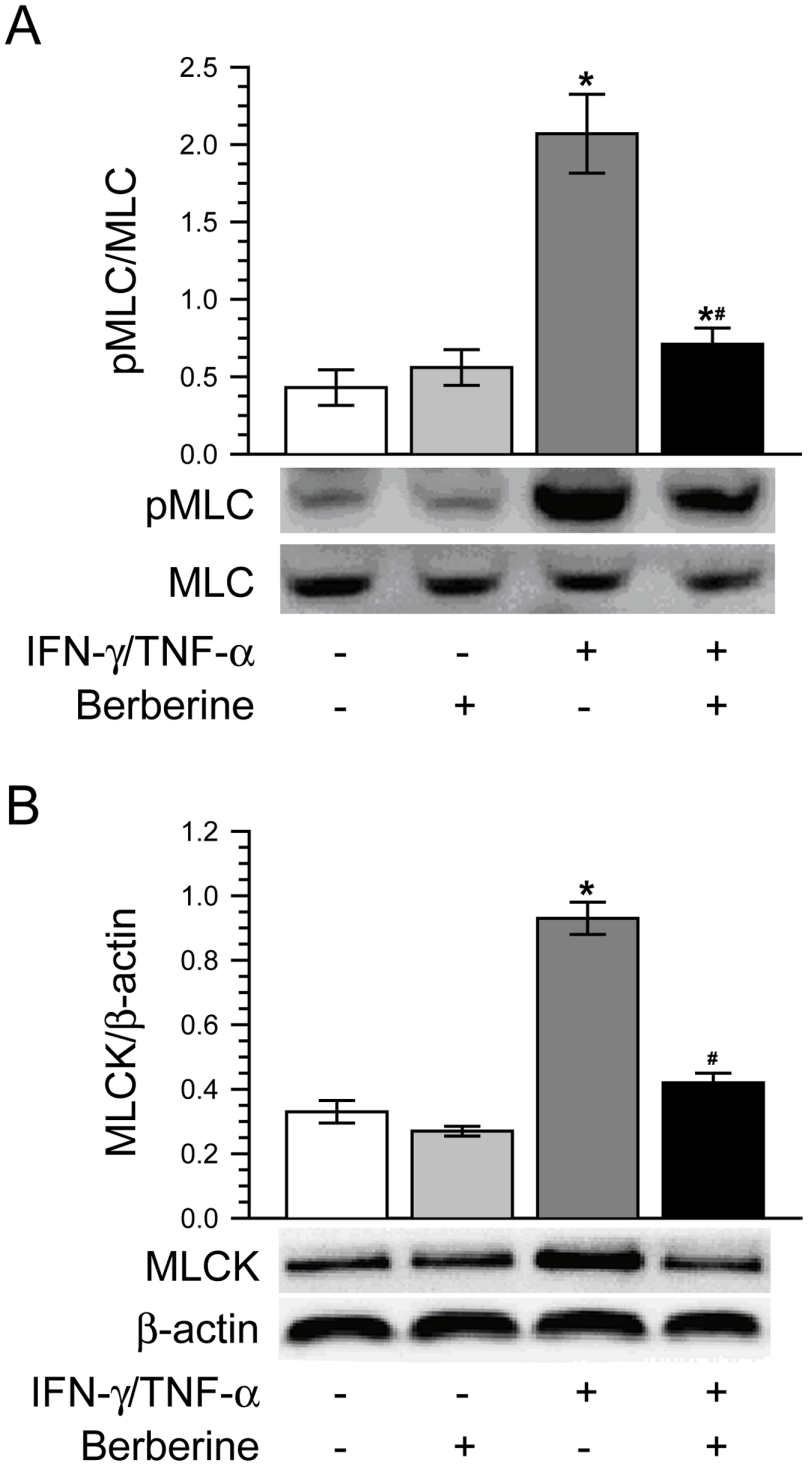
Berberine inhibits IFN-γ and TNF-α-induced increases of MLC phosphorylation and MLCK protein expression. Caco-2 monolayers were treated as described in Fig. 1A. A. Berberine significantly suppressed the increase of phosphorylated MLC expression induced by IFN-γ and TNF-α. *p<0.05, compared with control, #*p*<0.05, compared with IFN-γ/TNF-α. Data are representative of five similar experiments. B. Berberine abolished the IFN-γ and TNF-α-caused up-regulation of MLCK protein expression. *p<0.05, compared with control, #*p*<0.05, compared with IFN-γ/TNF-α. Data are representative of five similar experiments.

It is well recognized that MLCK-mediated phosphorylation of MLC plays a very important role in the physiological and pathophysiological regulation of intestinal epithelial tight junctions and paracellular leak pathways [1], [2]. Based on the above-mentioned remarkable protective effect of berberine on intestinal epithelial barrier function, we asked whether berberine alleviated IFN-γ and TNF-α-induced barrier dysfunction and tight junction disruption by blocking the increase of MLC phosphorylation. As shown in Fig. 4A, berberine alone had no significant effect on MLC phosphorylation in Caco-2 monolayers as compared with control. Treatment of Caco-2 monolayers with IFN-γ and TNF-α induced a significant increase of MLC phosphorylation, without markedly change in total MLC expression. Berberine treatment significantly attenuated the MLC phosphorylation increase elicited by IFN-γ and TNF-α.

MLCK is known to be the predominant determinant of MLC phosphorylation. Previous studies including ours have demonstrated that MLCK protein up-regulation is involved in barrier function disruption and paracellular hyperpermeability [9], [10], [15], [33], [34], [37]–[39]. Thus, we next investigated the effect of berberine on MLCK protein expression in Caco-2 monolayers challenged with or without IFN-γ and TNF-α. As demonstrated in Fig. 4B, like MLC phosphorylation, MLCK protein expression of Caco-2 monolayers was not significantly affected by berberine alone. After treatment of Caco-2 monolayers with IFN-γ and TNF-α, MLCK protein expression was significantly increased as compared with control. However, berberine treatment blocked the up-regulation of MLCK protein expression in Caco-2 monolayers treated with IFN-γ and TNF-α. It is suggested that berberine attenuates IFN-γ and TNF-α-induced intestinal barrier dysfunction by suppressing the MLCK-mediated MLC phosphorylation.

### Berberine Ameliorates IFN-γ and TNF-α-induced Barrier Dysfunction by Inhibiting HIF-1α Rather than NF-κB

**Figure 5:**
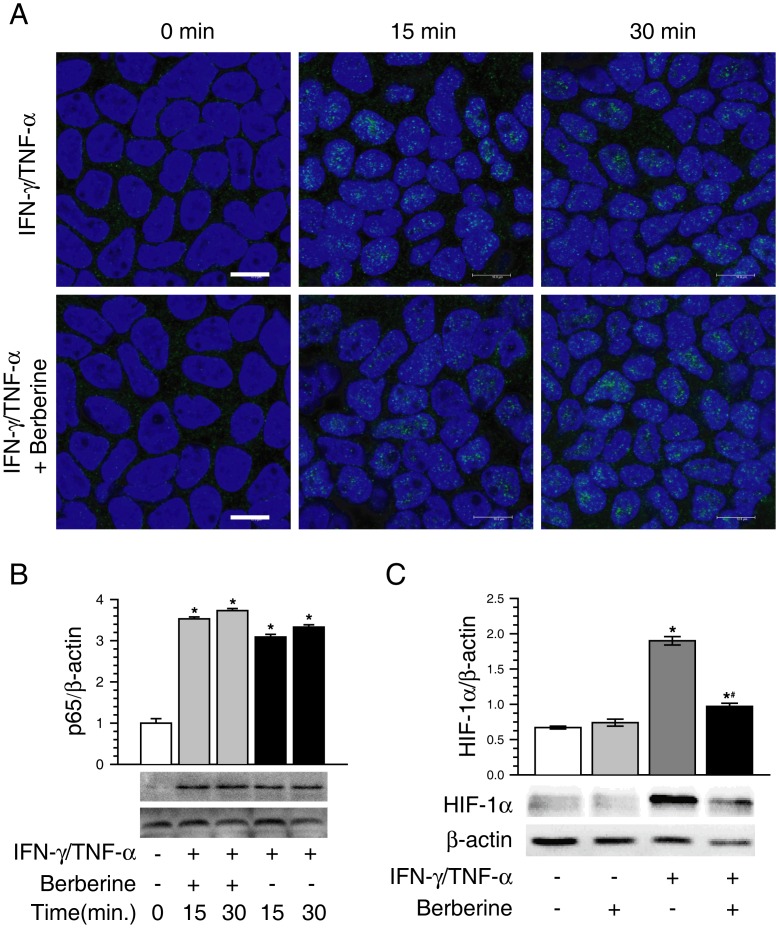
Berberine inhibits the activation of HIF-1α, but not NF-κB. A. Caco-2 monolayers were stained for NF-κB p65 by immunofluorescence. The nuclei were stained with DAPI. Treatment of Caco-2 monolayers with IFN-γ and TNF-α for 15 or 30 min dramatically induced NF-κB p65 accumulation within the nuclei. Berberine had no obvious effect on IFN-γ and TNF-α-elicited nuclear accumulation of NF-κB p65. Data are representative of three independent experiments. The green stands for NF-κB p65. The blue stands for nuclei. Scale bar = 10 µm. B. Treatment of Caco-2 monolayers with IFN-γ and TNF-α for 15 or 30 min increased nuclear NF-κB p65 significantly, whereas berberine treatment did not significantly change IFN-γ and TNF-α-induced increase of nuclear NF-κB p65. *p<0.05, compared with control (0 min). Data are representative of three similar experiments. C. Caco-2 monolayers were treated as described in Fig. 1A. Berberine treatment significantly inhibited IFN-γ and TNF-α-induced increase of HIF-1α protein. *p<0.05, compared with control, #*p*<0.05, compared with IFN-γ/TNF-α. Data are representative of five similar experiments.

Proinflammatory cytokines are known to activate nuclear transcription factor NF-κB. Previous studies have revealed that NF-κB activation is involved in barrier function disruption as well as MLCK up-regulation in proinflammatory cytokine-treated intestinal epithelial cells [11], [40], [41], and that berberine is able to inhibit NF-κB activation [23], [42]. Thus, based on the above results, we further determined whether NF-κB signaling pathway was involved in the protective effects of berberine on IFN-γ and TNF-α-induced intestinal barrier dysfunction, and MLCK up-regulation as well. As illustrated in Fig. 5A and B, treatment of Caco-2 monolayers with IFN-γ and TNF-α for 15 or 30 min elicited NF-κB p65 accumulation within the nuclei. Berberine treatment did not affect IFN-γ and TNF-α-triggered nuclear accumulation of NF-κB p65 in Caco-2 monolayers. This suggests that the protective role of berberine against IFN-γ and TNF-α-induced intestinal barrier dysfunction is NF-κB-independent.

Having excluded the involvement of NF-κB in the protective action of berberine on intestinal barrier function, we then sought to determine whether berberine attenuated IFN-γ and TNF-α-induced barrier dysfunction by inhibiting HIF-1α, because that HIF-1α was reported to mediate barrier function disruption in epithelial or endothelial cells [33], [34], [38], [43]–[47], and that berberine was shown to inhibit HIF-1α protein expression stimulated by hypoxia [48], [49]. As shown in Fig. 5C, berberine alone had no significant effect on HIF-1α protein expression when compared with control. After stimulation with FN-γ and TNF-α, HIF-1α protein expression was significantly increased as compared with control. The FN-γ and TNF-α-induced increase of HIF-1α protein expression was significantly inhibited by berberine treatment.

## Discussion

It is well known that inflammatory bowel disease, including ulcerative colitis and Crohn’s disease, is a chronic recurring inflammation of the intestinal tract. Although the etiology and pathogenesis of inflammatory bowel disease are incompletely elucidated, it is well recognized that it is characterized by the overproduction of a broad array of proinflammatory cytokines within the mucosa as well as the disruption of epithelial barrier function. Whether intestinal barrier dysfunction in inflammatory bowel disease is a primary contributor to mucosal inflammation or a consequence of the action of proinflammatory cytokines is still under debate. A number of research group, including ours, have demonstrated that proinflammatory cytokines disrupt intestinal epithelial barrier function both *in vitro* and *in vivo* [9], [10], [15], [33]–[37]. Thus, targeting the restoration of intestinal barrier function is still a worthwhile therapeutic strategy in acute or chronic enteropathies.

In this study, we show that berberine attenuates intestinal epithelial barrier dysfunction caused by proinflammatory cytokines *in vitro*, as evidenced by that berberine alleviates both TER decrease and paracellular permeability increase, and preserves the morphological distribution of tight junction proteins ZO-1, occludin and claudin-1 in Caco-2 intestinal epithelial monolayers exposed to simultaneous IFN-γ and TNF-α treatment. Berberine has been used in the treatment of gastroenteritis and infectious diarrhea for thousands of years. It has been demonstrated that berberine has an anti-inflammatory action both *in vivo* and *in vitro* [20]. Berberine has also been reported to be effective in ameliorating experimental colitis in rodents [23], [26]–[28]. Recently, several groups have shown that berberine protects epithelial or endothelial barrier function *in vitro* [29]–[31]. Similar to the findings of our present study, berberine has been revealed to attenuate barrier function disruption in intestinal epithelia exposed to TNF-α alone or sequential IFN-γ and TNF-α treatment [32], [50]. It is worth noting that our present study also shows that berberine causes a small increase in TER, but has no effect on paracellular FITC-dextran flux in untreated control Caco-2 monolayers, which is similar to previous study in HT-29/B6 intestinal epithelial monolayers [32], indicating that berberine may have an additional effect to tighten small ion flux in the absence of proinflammatory cytokines.

The molecular mechanism by which berberine ameliorates the intestinal epithelial barrier dysfunction induced by proinflammatory cytokines is currently unknown. Previous studies from several research groups, including ours, have demonstrated MLC phosphorylation mediated by the up-regulation of MLCK protein expression, which is triggered by the increased MLCK mRNA transcription, is required for the intestinal barrier defects induced by proinflammatory cytokines [9]–[11], [13], [15], [37], [40], [41]. Here, in this study we demonstrate that berberine inhibits the increases of both MLC phosphorylation and MLCK protein expression in Caco-2 monolayers exposed to simultaneous IFN-γ and TNF-α. Similarly, a recent *in vivo* study has revealed that berberine ameliorates the disruption of intestinal epithelial tight junction barrier by down-regulating MLCK pathway in a mouse model of endotoxinemia [51]. Thus, it is suggested that inhibition of MLC phosphorylation pathway mediated by the up-regulation of MLCK protein expression might be the molecular mechanism by which berberine attenuates intestinal barrier dysfunction caused by proinflammatory cytokines, though other potential mechanisms need to be further investigated. In fact, tyrosine kinase Src, Akt and NF-κB signaling pathways have recently been reported to be involved in the barrier-preserving effects of berberine in HT-29/B6 human colon monolayers treated with TNF-α [32].

It has been demonstrated that TNF-α-induced increase in intestinal epithelial tight junction permeability was mediated by NF-κB activation [52], and that NF-κB contributes to the transcriptional up-regulation of MLCK in Caco-2 intestinal epithelial cells challenged with proinflammatory cytokines [11], [40], [41]. In contrast, previous study has revealed that NF-κB activation is not an intermediate in TNF-α-induced barrier dysfunction in Caco-2 monolayers [9]. Although berberine has been reported to be capable of inhibiting NF-κB activation both *in vitro* and *in vivo* [23], [32], [51], in this study we show that berberine can not suppress the translocation of NF-κB p65 into the nuclei in Caco-2 monolayers treated with simultaneous IFN-γ and TNF-α. Thus, it is indicated that NF-κB signal pathway is not involved in the mechanism by which berberine attenuates the IFN-γ and TNF-α-induced barrier dysfunction in Caco-2 monolayers.

Previous studies have shown that HIF-1α plays a very important regulatory role in the inflammation responses [53]–[55], and that proinflammatory cytokines induce the expression of HIF-1α in enterocytes [56]. Some recent studies have demonstrated that HIF-1α activation contributes to the impairment of barrier function in vascular endothelia or intestinal epithelia [43]–[47]. Our previous studies have shown that inhibiting HIF-1α expression with specific HIF-1α inhibitor YC-1 or oligomycin attenuates the IFN-γ and TNF-α-caused barrier dysfunction by suppressing MLCK-mediated MLC phosphorylation in Caco-2 monolayers [33], [34]. In this study, we present that berberine inhibits the induction of HIF-1α protein in Caco-2 monolayers treated with IFN-γ and TNF-α. This is in accordance with the previous finding that berberine suppresses HIF-1α protein expression [48], [49]. Thus, taken together, it is speculated that inhibiting HIF-1α activation, at least in part, might be the molecular mechanism involved in the protective action of berberine against intestinal epithelial barrier dysfunction induced by IFN-γ and TNF-α.

In conclusion, our present study indicates that berberine attenuates intestinal barrier dysfunction elicited by IFN-γ and TNF-α *in vitro*. In addition, our data, for the first time, demonstrate that berberine inhibits the IFN-γ and TNF-α-induced up-regulation of MLC phosphorylation mediated by MLCK and HIF-1α, which might be the molecular mechanism involved in the protective action of berberine against intestinal epithelial barrier dysfunction triggered by proinflammatory cytokines.
